# 0450. Systemic dysregulation of the angiopoietin-1/2 system in adults undergoing cardiopulmonary bypass (CBP)

**DOI:** 10.1186/2197-425X-2-S1-O13

**Published:** 2014-09-26

**Authors:** E Charbonney, E Wilcox, Y Shan, P Perez d'Empaire, A Dugal, M Glogauer, GD Rubenfeld, S Sutherland, C Lilles, C Dos Santos

**Affiliations:** Université de Montréal, Montréal, Canada; University of Toronto, Toronto, Canada; Cleveland Clinic Foundation, Cleveland, USA; University of Washington, Seattle, USA

## Introduction

Systemic capillary leak syndrome after cardiopulmonary bypass (CBP) is a well-known phenomenon, accompanied by interstitial fluid accumulation and inflammation, and can lead to end-organ failure or increased hospital length of stay (LOS). The pathophysiology responsible for capillary leak involves activation of inflammation, complement, and coagulation. The endothelium plays a central role in the activation of coagulation, and activated/dysregulated endothelium contributes to microvascular leak and enhanced adhesion of leukocytes. However, the characterization and measurement of endothelial dysregulation is not well established. Angiopoietin-1 (Ang-1) and -2 (Ang-2) are molecules implicated in angiogenesis and regulation of endothelial permeability. Their differential changes of expression could be a surrogate to quantify endothelial dysregulation after CBP.

## Objectives

To characterize changes (delta) of Ang-1 and Ang-2 over time after elective CBP surgery in adults, and investigate conditions associated with measured level changes.

## Methods

Patients > 18 years, undergoing elective cardiac surgery using CBP, were enrolled. Baseline blood samples were obtained before surgery (T0), at admission to the ICU (T1) and the day following admission to the ICU (T2). Plasma Ang-1/-2 were measured using an ELISA and cytokines were measured using ELISA-based multiplex technology®.

## Results

Forty-one adult patients were enrolled consecutively, after obtaining consent.

Serum Ang-2 and Ang-2/Ang-1 ratio increased significantly over time (all p< 0.0001), while Ang-1 decreased (p=0.012). When compared to baseline, Ang-2 and Ang-2/Ang-1 ratio were significantly higher at both post-CBP time (T1 vs T0 and T2 vs T0; all p< 0.0001). IL-1β, IL-6, MCP-1, IL-10, IL-12 and IL-1RA rose significantly at T1. No meaningful correlation was found between changes in Ang-1/2 and cytokines.

A positive correlation was found between delta creatinine T0-T1 and delta T0-T1 for the ratio Ang-2/Ang1 (r= 0.38; p= 0.027). The hospital LOS correlated highly with delta T0-T2 for Ang-2 (r= 0.590; p< 0.0001) in patients with LOS ≤ 14 days (90%). The predictors of delta Ang-2 in multivariate analysis were female gender, ACE inhibitor use, blood transfusion and clamping time.

## Conclusions

CBP was associated with increases in serum Ang-2 and Ang-2/Ang-1 ratio, indicating endothelial activation and dysfunction post-CBP. Ang-1/2 dysregulation correlated with hospital length of stay and delta creatinine. Factors predicting endothelial dysregulation need to be further investigated.
